# Use of a Single-Item Ecological Momentary Assessment to Measure Daily Exercise: Agreement with Accelerometer-Measured Exercise

**DOI:** 10.3390/s24030946

**Published:** 2024-02-01

**Authors:** Kevin Sundquist, Joseph E. Schwartz, Matthew M. Burg, Karina W. Davidson, Keith M. Diaz

**Affiliations:** 1 Capital Rx, 1 World Trade Center, New York, NY 10007, USA; ksundquist@cap-rx.com; 2 Department of Psychiatry and Behavioral Science, Stony Brook University, Stony Brook, NY 11794, USA; jes2226@cumc.columbia.edu; 3 Center for Behavioral Cardiovascular Health, Columbia University Medical Center, New York, NY 10032, USA; 4 Department of Medicine, Yale University School of Medicine, New Haven, CT 06520, USA; matthew.burg@yale.edu; 5 Institute of Health System Science, Feinstein Institutes for Medical Research, Northwell Health, New York, NY 11030, USA; kdavidson2@northwell.edu; 6 Donald and Barbara Zucker School of Medicine at Hofstra/Northwell, Northwell Health, Hempstead, NY 11549, USA

**Keywords:** physical activity, exercise, ecological momentary assessment, smartphone, self-report

## Abstract

Accelerometers have been used to objectively quantify physical activity, but they can pose a high burden. This study was conducted to determine the feasibility of using a single-item smartphone-based ecological momentary assessment (EMA) in lieu of accelerometers in long-term assessment of daily exercise. Data were collected from a randomized controlled trial of intermittently exercising, otherwise healthy adults (N = 79; 57% female, mean age: 31.9 ± 9.5 years) over 365 days. Smartphone-based EMA self-reports of exercise entailed daily end-of-day responses about physical activity; the participants also wore a Fitbit device to measure physical activity. The Kappa statistic was used to quantify the agreement between accelerometer-determined (24 min of moderate-to-vigorous physical activity [MVPA] within 30 min) and self-reported exercise. Possible demographic predictors of agreement were assessed. Participants provided an average of 164 ± 87 days of complete data. The average within-person Kappa was **κ** = 0.30 ± 0.22 (range: −0.15–0.73). Mean Kappa ranged from 0.16 to 0.30 when the accelerometer-based definition of an exercise bout varied in duration from 15 to 30 min of MVPA within any 30 min period. Among the correlates examined, sex was significantly associated with agreement; mean agreement was higher among women (**κ** = 0.37) than men (**κ** = 0.20). Agreement between EMA self-reported and accelerometer-measured exercise was fair, suggesting that long-term exercise monitoring through a single-item EMA may be acceptable.

## 1. Introduction

Physical activity is widely recognized to be important for the prevention and treatment of a myriad of chronic diseases and is a key contributor to healthy aging and quality of life [1,2,3]. The importance of physical activity as a key lifestyle factor [1,2,3] has necessitated the need to develop methodologies that accurately measure habitual volumes and patterns of physical activity. Currently, habitual physical activity is most commonly measured in two ways: (i) self-report, where physical activity over the past week/month/year is queried or (ii) objective measurement via accelerometry over a short-term period (e.g., 7 days) where the observed physical activity levels are inferred to be indicative of “habitual” activity [4,5,6,7]. Both methods have their advantages and limitations. Self-report can be prone to substantial reporting/measurement error [8] which, in part, has been attributed to challenges with physical activity recall and social desirability bias. Studies that use an accelerometer to more objectively quantify physical activity and overcome the limitations of self-report are conventionally short in length, often 7–14 days [4,5,6,7,9]. Although such accelerometer protocols are advantageous for determining an individual’s regular or habitual level of physical activity, they do not allow researchers to assess week-to-week and month-to-month changes in physical activity and influencing factors.

Barriers to the long-term study of physical activity (e.g., 1–12 months) using accelerometers include participant burden, logistical challenges, and analytic complexities related to processing large volumes of raw data. Research-grade accelerometer devices have a limited storage capacity and a typical charge duration of 14–45 days. For long-term studies, periodic switching out of the accelerometer devices is thus required, which can be impractical for larger studies. More consumer-centric devices, such as those made by Fitbit, have been validated [10,11] and can overcome the limitations of research-grade accelerometers, primarily because data can be transmitted wirelessly and devices can be charged by the user. However, such devices still impose substantial participant burden (daily device wear, regular charging, data syncing) for the resulting data to be truly comprehensive. With the rise of smartphones and their exponential capabilities, daily activity tracking through “apps” or text messages provide the potential to overcome limitations of available approaches for the long-term measurement of physical activity [12]. For example, a brief single-item measure administered at the end of each day which queries whether an individual exercised on a given day can be easily integrated into smartphone apps/devices. By permitting the collection of daily, end-of-day self-reports of physical activity in real time (thus, not asking the individual to mentally “compute” or estimate their amount or frequency of exercising), we can study daily activity with relatively low participant burden. Early evidence suggests high compliance (>80%) over short-term periods (4–7 days) assessing physical activity via text message [6]. Thus, with potentially high response rates, smartphone-designed daily surveys could have a high yield of data over a lengthy study period at a low cost.

The purpose of this study was to examine the level of within-person agreement between a single-item, smartphone-based, daily EMA report of exercise and accelerometer-defined exercise in a cohort of 79 ambulatory adults studied for 365 consecutive days. Our analysis will aid in determining the feasibility of using smartphone-based ecological momentary assessment (EMA) for the long-term (e.g., 1 year) assessment of health-enhancing physical activity (e.g., exercise) in lieu of accelerometers. As a secondary purpose, we assessed person-level factors associated with individual differences in the level of agreement between EMA and accelerometer defined exercise.

## 2. Material and Methods

**Study Design:** This study was a randomized controlled experiment conducted with 12 months of observation that was designed to develop and deliver a personalized physical activity intervention utilizing mobile health technologies [13]. A general sample of adults who were self-reported intermittent exercisers (described below) and who did not have any medical contraindications to physical activity were enrolled. After enrollment, participants completed a battery of questionnaires at baseline and monthly thereafter for the duration of the study. Participants were asked daily about their physical activity through smartphone-based EMA. Accelerometer data were also collected continuously for the 12-month study period. After the first 6 months of observation, participants were randomized to either an intervention group that received their activity fingerprint (that is, a personalized description of those factors that significantly predicted whether they exercised or not on a given day) or a control group that received general descriptive information about their exercise levels. Thereafter, both groups completed an additional 6 months of observation. This study was conducted at Columbia University Medical Center (CUMC) and approved by its institutional review board. Access to the study dataset and information about this study’s execution and materials are publicly available at https://osf.io/kmszn.

**Study Participants:** We recruited a convenience sample of 79 adults through advertising at CUMC. Participants were English speaking, adults, self-reported intermittent exercisers (self-reported exercising [of any type] at a moderate or vigorous intensity 6–11 times per month, but not on a regular basis), had daily access to a computer with Internet, and had an iPhone or Android phone. We excluded individuals who had been recommended to restrict physical activity, were deemed unable to comply with the protocol, were unavailable for 12 continuous months, had serious medical problems affecting their ability to engage in physical activity, had an occupation that demanded rigorous activity or would make responding to the EMA dangerous, or were unable to read and speak English. 

**Figure 1:**
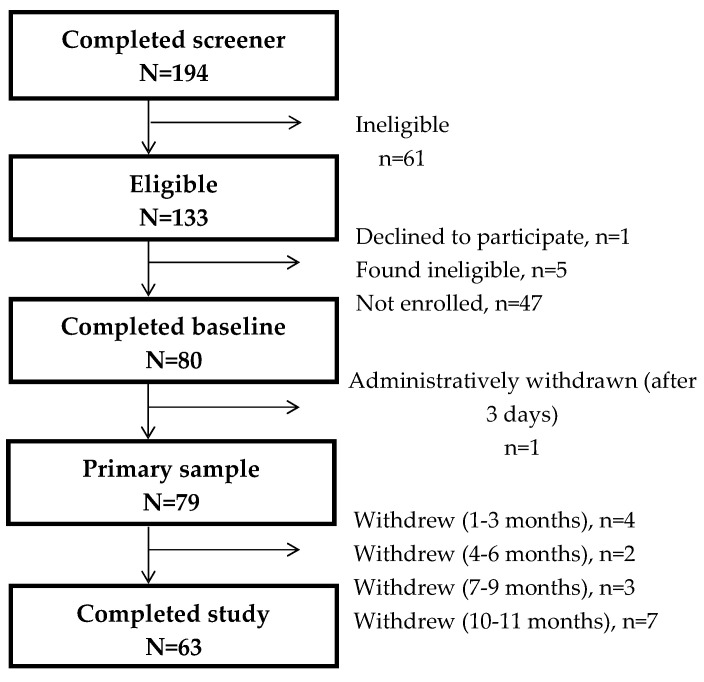
Consort diagram showing the inclusion/exclusion of participants for analysis.

Overall, 194 individuals completed the online screening, of whom 61 were found ineligible. Of the remaining 133, 80 individuals completed the baseline run-in period, with 1 being administratively removed, leaving a final study sample of 79 participants (Figure 1). With 6 participants withdrawing prior to 6 months and 10 withdrawing prior to 11 months, a total of 63 participants fully completed the study (>11 months of data). Primary analyses included all 79 participants.

**Accelerometer PA:** Physical activity was continuously monitored using a wrist-worn model of the Fitbit (Fitbit Flex, San Francisco, CA, USA). The Fitbit Flex is a microelectromechanical triaxial accelerometer that tracks the wearer’s daily physical activity including steps, intensity of activity (sedentary, light, moderate, or vigorous), and energy expenditure. We and others have shown that the Fitbit Flex is a valid and reliable device for measuring physical activity in adults [10,11]. Data from the device automatically uploaded to the Fitbit website whenever the device was within 15 feet of the base station, which was plugged into the participant’s own Mac or PC computer. Participants were instructed to sync and charge their device at least every 5–7 days to ensure no loss of activity data. The Fitbit Flex was chosen for the study because it does not require the participant to return to the study office for syncing or battery charging. 

Minute-by-minute activity data were extracted using Fitabase (Small Steps Labs, San Diego, CA, USA). We defined a “social day” as the period from 3 a.m. until 2:59 a.m. the next day. Non-wear was defined as >60 consecutive minutes with fewer than 10 steps. Only days with ≥10 h of wear time were considered “valid” and included in analyses [14]. Each valid day was classified as an exercise or non-exercise day; defined objectively as at least 24 min of moderate or vigorous physical activity (MVPA) within any consecutive 30-min period, thereby allowing up to 6 min of below-threshold physical activity (e.g., rest) during the exercise period. This definition was adapted from conventional accelerometer-processing approaches used in many population-based studies wherein a healthful bout of physical activity is defined as a 10 min or longer bout of MVPA with an allowance of 2 min below threshold (e.g., 8 out of 10 consecutive minutes) [15,16,17]. 

**Self-reported exercise:** Participants were asked to complete five EMA surveys each day (morning, evening, and three random mid-day surveys) for the entire 12-month study period. Participants were sent text messages on their own iPhone or Android phone containing a unique URL link which expired after 70 min. The system tracked when the notification went out and when the participant started/completed the assessment. If an instance of the survey was closed before finishing, participants were able to return to the survey via the URL to complete it. As part of the daily EMA surveys, each evening participants completed an end-of-day report that included 3 items, including 1 item where they were asked to respond (Yes or No) to the question “Did you exercise today for 30 min or more at a moderate or vigorous level”. This validated single-item physical activity instrument was designed to ascertain adherence to physical activity guidelines [18].

**Correlates:** Demographic factors (e.g., age, sex, etc.), caregiver status, body mass index (BMI), perceived stress, and health-related technology use were examined as potential correlates of the participant-specific agreement between the accelerometer and self-report measures. Demographic factors and caregiver status were assessed via a self-administered questionnaire at baseline. Technology use for health-related purposes was also assessed at baseline with the question “In a typical week, do you use “apps” on a smartphone for health purposes (e.g., diet, sleep, exercise)”. Perceived stress was assessed by Cohen’s Perceived Stress Scale (PSS) [19] at baseline and monthly thereafter. The average perceived stress across study visits was quantified and examined as a potential correlate. The number of exercise days (expressed as a percentage of valid days) according to accelerometry and self-report and their squared terms (to capture non-linearity) were also examined.

**Statistical Analysis:** The kappa statistic was used to quantify the agreement between the daily dichotomous end-of-day exercise question and the same-day accelerometer-derived measure of exercise (yes/no) for each participant. For primary analyses, our accelerometer-based definition of exercise was at least 24 min of MVPA within any 30 min period (see above). As a secondary analysis, we explored the effect that changing the 24 min criterion to alternative values, ranging from 15–30 min, had on the average agreement. Additionally, we examined the level of agreement when the accelerometer definition included all physical activity intensities (light, moderate, or vigorous) or exclusively vigorous physical activity. Linear regression analyses were conducted to assess which person-level factors were associated with the level of agreement between the self-reported and accelerometer measures of exercise. Univariate analyses were initially conducted, with subsequent multivariate analyses conducted for all significant (*p* < 0.05) person-level factors from univariate models. Multivariate models included adjustment for all tested correlates simultaneously.

The above analyses IIed all 12 months of observation. As a sensitivity analysis, all analyses were repeated restricted to the first 6 months of observation, i.e., the pre-randomization period. Furthermore, as noted above, all analyses were repeated varying the accelerometer definition of exercise by time (15 to 30 min within a 30 min period) and intensity (light, moderate, or vigorous activity; vigorous activity only). All analyses were conducted in SAS version 9.4 (SAS Institute, Cary, NC, USA).

## 3. Results

**Participant Characteristics:** Among the 79 participants, the mean age was 31.9 ± 9.5 years, 57.0% were female, 13.9% were black, 27.8% were Hispanic, and 40.5% had a graduate degree or higher (Table 1). Compliance to self-report (i.e., end-of-day response) and accelerometer wear (e.g., ≥10 h of wear) were 64% and 68%, respectively, over an average of 323 days of active study participation. Across all participants, there was a total of 12,807 person-days wherein both self-reported and accelerometer-assessed exercise data were available. The median number of days with complete data at the participant level was 164 ± 87. The mean percentage of days exercised, based on self-report and accelerometer data, was 37.5 ± 22.6% and 32.2 ± 16.5%, respectively.

**Figure 2:**
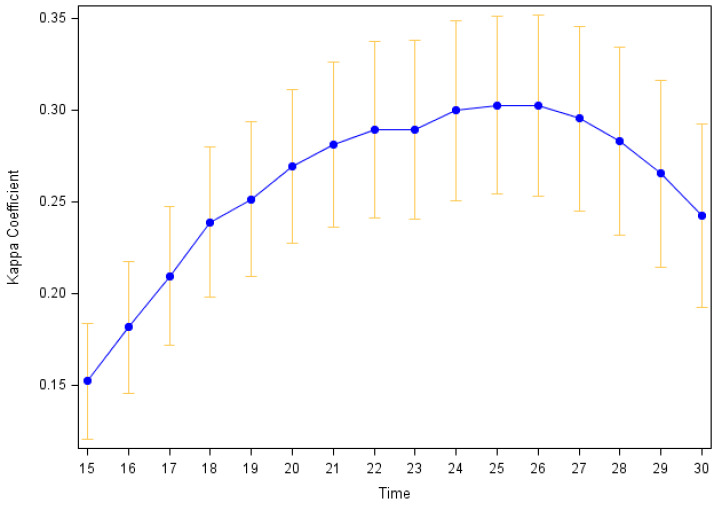
Average agreement between self-reported and accelerometer-determined exercise by number of minutes of MVPA required to define an exercise bout. Median Kappa with 95% confidence intervals. The definition of an accelerometer-determined exercise day ranges from 15 to 30 min of MVPA within any 30 min period.

**Figure 3:**
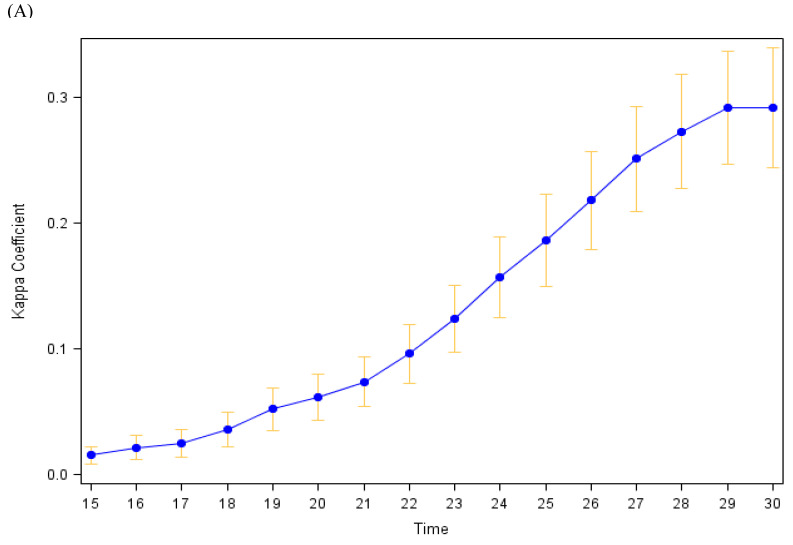

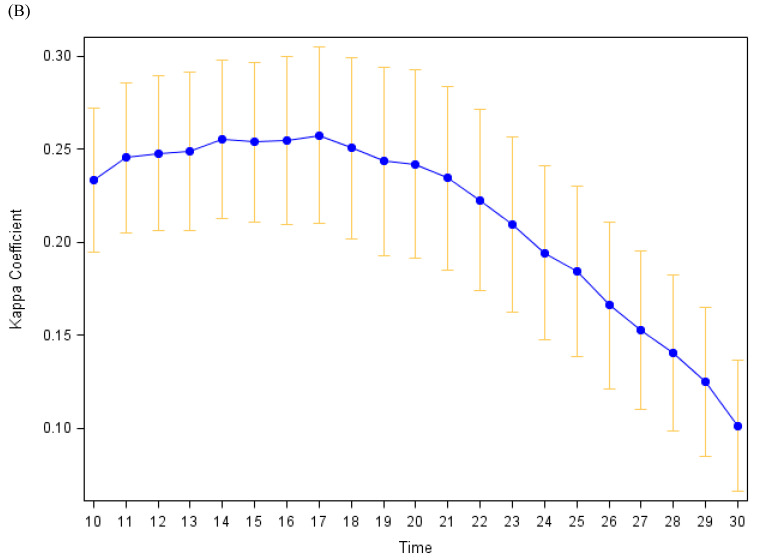
Average agreement between self-reported and accelerometer-determined exercise by level of physical activity intensity classified as exercise. (**A**): All physical activity intensity categories (light, moderate, or vigorous) used to define exercise. (**B**): Only vigorous physical activity defined as exercise. Median Kappa with 95% confidence intervals. The definition of an accelerometer-determined exercise day ranges from 15 to 30 min of physical activity (all intensity categories or only vigorous intensity) within any 30 min period.

**Agreement between self-reported and accelerometer-measured exercise:** Participants’ individual Kappa values ranged from −0.15 to 0.73, with a mean of 0.30 (SD = 0.22) using our primary definition of accelerometer-measured exercise (24 min of MVPA within any consecutive 30 min period). The pooled Kappa (**κ_p_** ± SE) across all 12,807 observations was **κ_p_** = 0.36 ± 0.01. Figure 2 shows how the mean participants’ Kappa values varies as the definition of accelerometer-measured exercise is changed from 15 to 30 min of MVPA within a 30 min period. The 24–26 min (of 30 min) definitions yielded the highest mean Kappa (**κ** = 0.30); with the mean Kappa decreasing with both higher and lower thresholds for the required number of minutes of MVPA. Figure 3 shows similar plots when the level of intensity that is considered exercise includes all activity intensities (e.g., light, moderate, and vigorous; top panel) or just vigorous intensity activity (bottom panel). The maximum Kappa (all intensities: 0.29 ± 0.02; vigorous intensity: 0.26 ± 0.02) had only minor fluctuations in value with differing intensity definitions, yet they occurred for different bout duration criteria (29 or 30 min of activity when all intensities are included vs. 17 min of vigorous activity when only vigorous activity is considered). In sensitivity analyses restricting the analysis period to the first 6 months of observation, results were similar.

**Correlates of Agreement:** Only sex was associated with agreement between accelerometer-measured (24 out of 30 min of MVPA) and self-reported exercise in univariate analyses (Table 2). In multivariate adjusted models, sex remained significantly associated with level of agreement (β = 0.21 [SE = 0.05]; *p* < 0.001). Females, on average, had a higher Kappa than males (0.37 ± 0.22 vs. 0.20 ± 0.19). When the definition of accelerometer-measured exercise was varied by active duration criteria and intensity criteria, females continued to exhibit a significantly higher level of agreement with the daily self-reports of exercise. In sensitivity analyses restricting the analysis period to the first 6 months of observation, results were similar.

## 4. Discussion

The primary purpose of this study was to evaluate the utility of using a single-item, end-of-day, smartphone-based EMA to ascertain engagement in physical exercise as compared to a validated accelerometer measure. In this cohort of 79 healthy adults where exercise participation was assessed daily for 1 year via both a smartphone-based, single-item EMA question and accelerometry, we found that the average agreement between the two daily exercise measures was fair (**κ** = 0.30) [20]. The examination of factors potentially associated with the level of agreement between the EMA and accelerometer measures of exercise showed that sex was a robust correlate of agreement, with females exhibiting a greater level of agreement than males. 

As the broad public adoption and maintenance of a physically active lifestyle remains elusive (24% of US adults meet current physical activity guideline recommendations) [21], a key public health imperative is to identify—and then intervene on—the factors that serve as obstacles to the adoption and maintenance of physical activity. Engagement in physical activity varies from day to day, week to week, and month to month in response to environmental factors (e.g., weather) and everyday life exposures (e.g., job strain, financial/time pressures, caregiver responsibilities) [22]; thus, the elucidation of factors that influence physical activity participation is complex and has been largely limited by methodologies (e.g., self-report, 7-day accelerometer protocols) that typically capture physical activity over relatively short periods. In the present study, we examined the utility of smartphones to measure long-term physical activity using a simple end-of-day text message that queries (yes or no) engagement in 30 min of exercise on a given day. In the US, 85% of adults own a smartphone [23] and thus the potential of using smartphone-based tracking of long-term physical activity patterns (and the factors that influence these patterns) may be sizable, practical, and economical. Over a mean enrollment period of 323 days, we observed a 64% completion rate (~214 days/person) of the single-item EMA. Previous studies that examined physical activity levels via smartphone-based EMA have reported compliance rates of 30–80% over the course of four to seven days [4,6]. Although the compliance rate in our study could be considered relatively low, in light of the fact that most accelerometer studies require only 4 valid days over a 7-day wear period to be considered valid (~57% of total days), the collection of over 200 days of self-report data is substantial and highlights a potential strength of using end-of-day text message prompts to capture long-term physical activity patterns.

In a systematic review of 148 studies, Prince et al. [8] examined the extent of agreement between self-report and direct measures of physical activity in adults, concluding that there was generally low-to-moderate agreement between self-report and accelerometer measures. Thus, our findings are consistent regarding the discrepancy between self-report and accelerometer measures of physical activity. Notably, the discrepancy between self-reported and accelerometer-measured physical activity have also been reported in the National Health and Nutrition Examination Survey (NHANES) [24]. Comparisons of self-reported adherence to physical activity recommendations with those directly measured by an accelerometer indicated that self-reported rates of exercising were much higher than those measured by accelerometers [24], which authors attributed to respondents misclassifying sedentary or light activity as moderate or from underestimations of activity duration by the accelerometers. Both factors could have contributed to the only fair level of agreement in the present study, along with conventional limitations of self-report measures, including recall and social desirability bias. While EMA is designed to minimize recall bias, it does not eliminate it and cannot account for social desirability bias. Furthermore, it is important to recognize that wrist-based accelerometers are unable to capture certain types of activities, such as swimming (the Fitbit Flex model used in the present study was not waterproof and had to be removed), stationary cycling, and muscle-strengthening activities. Thus, the misclassification of these types of physical activities by the comparative standard could also have contributed to the fair levels of agreement observed. The observed agreement must be interpreted bearing in mind that accelerometers may not measure all activities that an individual would self-report as exercise and are intended to measure ambulatory movement (not evaluate exercise behavior per se) [25]. This is especially pertinent to wrist wear locations, which are subject to greater movement artifact and misclassification errors than hip or thigh wear locations. As such, the fair agreement observed may be a result of weaknesses in both instruments and underscores that each measure may capture different types/aspects of exercise behavior and thus a triangulation approach for measuring physical activity that leverages both methods may be optimal. 

A 2022 systematic review identified 10 studies that have evaluated the validity of EMA-based assessments of physical activity [26]. The EMA methods in the identified studies widely varied. Six studies used real-time prompts (i.e., “What are you doing right now?”) while four used retrospective prompts (i.e., “How many minutes have you been physically active since the last prompt?”). The number of prompts ranged from 1 to 72 per day, with the majority of studies (7 out of 10) utilizing 6–8 prompts per day. Most studies (6 out of 10) used one-item prompts. Several studies (6 out of 10) did not report statistics that characterized the magnitude of agreement between EMA and criterion measures. Thus, the ability to draw conclusions regarding the validity of existing EMA-based measures of physical activity was determined to be limited [26]. Only two studies among adult samples reported statistics to discern level of agreement. Among a sample of college students, two-item real-time prompts (“What are you doing right now”; if physical activity was selected, then intensity was queried) delivered 7 times per day had match rates of 59%, 22%, and 4% for light-, moderate-, and vigorous-intensity physical activity, respectively, relative to hip-mounted Actigraph accelerometer measures [7]. Knell et al. reported that a single, retrospective EMA prompt delivered in the morning querying the number of minutes of MVPA in the previous day had a Lin’s concordance correlation of 0.28 with MVPA minutes via hip-mounted Actigraph accelerometer among a general sample of adults [25]. As challenges have been documented with recall of intensity-specific minutes of physical activity, given the limitation of not incorporating the context of physical activity (since the health benefits of occupational physical activity are not well established [27]), and considering that real-time prompts (“What are you doing right now?”) bear the risk of missing physical activity episodes, our EMA-based physical activity measure was designed to minimize recall errors/challenges related to quantifying MVPA minutes, specifically target health-enhancing physical activity (rather than incidental or occupational physical activity), and be retrospective in nature. As such, our findings add to a largely scant evidence base on the validity of EMA-based assessment of physical activity.

The utility of our EMA-based physical activity must be considered in the context of the fair agreement observed with accelerometer measures. The present EMA protocol was designed with a goal of developing a tool to study the time-varying dynamics of exercise behavior and its antecedents over long-term periods, as well as assess treatment effects on exercise habits in a long-term trial. The fair agreement observed warrants caution in the application of our EMA protocol as it may lack sufficient sensitivity to evaluate meaningful short-term changes. If a measure is only capturing some, but not all exercise, it may not be sensitive enough to capture treatment effects, especially in short-term studies where only a small number of exercise bouts occur [26]. The necessary sensitivity of any EMA protocol will depend on the specific study design, including length of study and frequency of exercise. It should be noted that we have previously reported on the randomized controlled experiment component of this study and showed that our EMA protocol was sensitive enough to detect significant changes (8% change in days exercised) over a 3-month time frame [13]. However, it is unclear whether our EMA protocol would be sensitive enough for shorter-term studies. Nonetheless, the use of accelerometers is not always feasible due to expense, poor compliance with device wear, and/or practical constraints for researchers/participants. As EMA is less susceptible to recall bias and has been demonstrated to be superior to self-report questionnaires [25], EMA-based methods may be a viable alternative in some circumstances.

The examination of factors associated with the level of agreement between the self-report and accelerometer measures of exercise indicated that sex was a robust correlate of agreement, with females having a greater level of agreement between the two exercise measures compared to males. This association between sex and level of agreement was evident with all accelerometer-based definitions of exercise. Our findings are consistent with previous studies which have reported that the agreement between self-report and objective/biochemical measures differs by sex for health-related behaviors including smoking [28], healthcare utilization [29], and physical activity [30]. Contributing factors to the observed sex differences in agreement between self-report and accelerometer-measured exercise are unclear; however, it has been hypothesized that it is more socially desirable for females to report healthful behaviors than males, thus they may exhibit better recall [31]. We can also not rule out that differences in agreement could be attributed to differences in the types of physical activities that males and females participate in. Previous studies have demonstrated that males are more likely to engage in muscle-strengthening activities, for which accelerometers have been reported to have lower accuracy at measuring [32,33]. Future studies examining factors that contribute to sex differences in the reporting and identification of physical activity may be warranted.

Several limitations should be noted when interpreting findings from the current study. First, the mean number of days with both EMA and valid accelerometer data was only moderate (~164 days). However, this is one of the longest EMA/accelerometer studies ever conducted, with over 12,000 person-days of data available for analysis. Further, as noted above, most accelerometer studies (including studies validating self-report) conventionally require only 4 valid days of accelerometry over a 7-day wear period to be considered valid. Second, we recruited a convenience sample of relatively healthy individuals, potentially limiting the generalizability of our findings. Nonetheless, the racial/ethnic diversity of the study sample (14% black, 28% Hispanic) improves its generalizability to these populations. Third, our single-item, end-of-day, smartphone-based EMA allowed for only a binary response (yes or no). It is unknown whether items that queried duration, intensity, or type of physical activity would have yielded different levels of agreement. Fourth, our sample entailed only 79 participants. While the collection of over 12,000 observation days was adequate to assess the level of agreement between self-reported and accelerometer-measured exercise, the analyses examining the person-level factors associated with the agreement between the EMA/accelerometer measures may be underpowered. Thus, caution is warranted when interpreting these results. Finally, participants were required to have a smartphone for study inclusion and further reported high levels of health-related technology use (50%). Thus, our study population may have been more likely to comply and be aware of the benefits of tracking daily exercise and its relation to health. Future studies in populations with limited smartphone exposure/usage (e.g., low SES) may be warranted. 

## 5. Conclusions

In this cohort of 79 healthy adults in whom exercise participation was assessed daily for 1 year via both a smartphone-based, single-item EMA approach and accelerometry, we found that agreement between self-reported EMA responses and accelerometer-measured physical activity was fair. These findings suggest that smartphone-based EMA for long-term physical activity monitoring in lieu of an accelerometer may be feasible and acceptable in that it has some, but not high, validity vis-a-vis accelerometer-assessed physical activity. With a completion rate of 63% (a median 214 days), end-of-day EMA of exercise participation via smartphone may have some utility as a tool for researchers examining the long-term patterns of physical activity. Future studies examining the feasibility and reliability of smartphone EMA compared to accelerometer measures of physical activity, especially in more diverse populations, are still needed. Evaluation of EMA reports that query more robust information concerning the type, duration, and intensity of physical activity is also needed.

## Figures and Tables

**Table 1:** Participant characteristics.

*Variable*	Mean ± Standard Deviation or % (n) (N = 79)
Age (years)	31.9 ± 9.5
BMI (kg/m^2^)	26.4 ± 5.3
Female sex, % (n)	57.0 (45)
Black race, % (n)	13.9 (11)
Hispanic ethnicity, % (n)	27.8 (22)
Graduate degree or higher, % (n)	40.5 (32)
Partner/spouse, % (n)	40.5 (32)
Caregiver (child or elder), % (n)	49.4 (39)
Average monthly reported stress	22.6 ± 6.0
Health-related tech use, % (n)	49.4 (39)
Valid days ^a^	162.1 ± 86.7
% of days self-reported exercise	37.5 ± 22.6
% of days objectively measured exercise	31.9 ± 16.5

^a^ Defined as ≥10 h of accelerometer wear and EMA response.

**Table 2:** Factors associated with the level of agreement between self-reported and accelerometer-measured exercise.

*Predictor Variables*	*β (StErr)*	*p-Value*
Age	0.00 (0.030)	0.95
BMI	−0.00 (0.004)	0.70
Female sex	0.17 (0.050)	<0.001
Black race	0.03 (0.073)	0.73
Hispanic ethnicity	−0.09 (0.055)	0.13
Graduate degree or higher	0.04 (0.051)	0.41
Partner/spouse	−0.02 (0.051)	0.71
Caregiver	0.02 (0.068)	0.73
Average monthly reported stress	0.00 (0.020)	0.89
Health-related tech use	0.07 (0.050)	0.15
% of days self-reported exercise	−0.01 (0.010)	0.42
% of days accelerometer-measured exercise	0.03 (0.015)	0.08

## Data Availability

Data are available publicly at https://osf.io/kmszn.
